# Everybody's Page

**Published:** 1891-05-09

**Authors:** 


### EVERYBODY'S PAGE.

The disease *Beri-beri*, which in Ceylon signifies weakness, and in Japan is called *Kakke*, is considered by Dr. Kakaki of the Japanese navy to be due in large measure to the use of food deficient in nitrogenous and fatty matters. This theory was put to the test of actual experiment in the navy, the food of the sailors being materially changed, and the result has been in Japan that the disease has almost disappeared in that service.

Opinions on Mr. Jacoby's now defunct Itinerant Street Musicians' Bill seem to vary; personally we are sympathetic with the promoter of the Bill to a certain extent. His antagonists plead the "liberty of the subject," "the pleasure of the masses," and so on. This is all very well as far as it goes, but who does not know the deathly groans of an itinerant cornet soloist or the tantalising effect on one's brain of a discordant German band? Surely this might be mitigated if, as we suggested last year, street musicians were obliged to be registered as possessing some small amount of musical knowledge, but perhaps we are "hyper-sensitive."

The story of the girl who has recently appeared at one of the police-courts, speaking an unrecognisable tongue, recalls to memory Dr. Wilkinson and the Princess Caraboo. An interesting looking woman called on a lady living at Clifton; her language was unintelligible, but by signs she made it understood that she had been shipwrecked, and was a princess in her own country. She wrote some gibberish, and signed it "Caraboo." The paper was sent to the Universities, but the authorities could not interpret it. Among the many savants who called on the princess was Dr. Wilkinson, a physician, who practised in Bath early in the present century. He took much interest in the case, and had almost succeeded in making a key to the language, when the bubble burst! And here the similarity between the two women ceases, as one proves to be a Lithuanian peasant, the other an ingenious native of Bristol named Mary Wilcox. Dr. Wilkinson was known ever afterwards as Dr. Caraboo. Peach, in his "Historic Houses of Bath," gives a very good account of the imposture.

Why is it that so many of our fellow-creatures, when they are walking abo it the town, will indulge in the practice of doing their best to poke out the eyes of, or otherwise maim, their fellow-pedestrians? Every day we notice men and women urging on their mad career with their umbrella or walking stick under their arm, the point well up ready to slay any unwary comer. Life may be a struggle, but it is scarcely a fair one if it is to be abruptly terminated by the ferrule of an umbrella.

For development of grace of figure and movement perhaps no exercise beats running. The old Greeks ran, and who were finer specimens of symmetry than they were? The active tribes of the American Indians, who have been runners from untold years, would put the average white man in the background in comparison of physical form. Running in moderation developes muscles where they ought to be, sends the shoulders back, gives balance, and keeps the feet in correct position. Next to running there is nothing to beat walking, but nearly all of us are prone to walk on our heels; we ought to spring forward like a modified edition of the picture of the gentleman in Waukenphast's boots, driving our body on with the back muscles of our legs.

If the "coming dress" is to look anything like the sketches of it in the *Daily Graphic* may the fates be merciful to us and keep such an affliction afar off just yet. Custom has made us like to see our womankind in skirts plain and simple. We do not feel we should appreciate our female friends in Wellington boots and a skirt that looks like the voluminous nether garment of a Zouave. We do not pine for Japanese, Turkish, or any other variety of clothing; all we want is to see women dressed rationally in the true sense of the word. Lately we notice dresses are made so long as to assist the vestries in the cleansing of the streets. Now a woman who will be the slave of fashion to that extent will never be well or sensibly dressed, and she will never listen to a Dress Association; it is husbands, fathers, and brothers who should raise a protest against such customs. Plain skirts made to escape the mud will survive Bloomer costumes, past, present, or future.

